# Letter to the Editor From Papadimitriou: “Beyond Stages: Predicting Individual Time-dependent Risk for Type 1 Diabetes”

**DOI:** 10.1210/clinem/dgaf029

**Published:** 2025-01-21

**Authors:** Dimitrios T Papadimitriou

**Affiliations:** Department of Pediatrics, University General Hospital of Larisa, Larisa 413 34, Greece

The Hippocratic Oath holds a prominent place in medical education: “I will use my power to help the sick to the best of my ability and judgment; I will abstain from harming or wronging any man by it,” remaining foundational to the ethics of medical practice, rooted in the logic of prevention over cure. These principles resonate strongly with predicting individual time-dependent risk for type 1 diabetes (T1D) (1), testing for islet autoantibodies for identifying early-stage disease with a diverse but lifetime risk (2), and the consensus guidance for monitoring individuals with islet autoantibody–positive prestage 3 T1D (3), advocating trial participation of “approved” therapies for stage 2 T1D. Hoping for the reversal of diabetes with our own stem cells (4), several questions merit consideration:

## Food and Drug Administration-approved Therapies: Teplizumab

Teplizumab poses significant concerns due to its potential side effects, including lymphopenia, leukopenia, cytokine storm, and sepsis (5). Prohibitively expensive, exclusive of broader medical and socioeconomic cost implications, it has limited clinical benefits; it may delay the initiation of insulin therapy for up to 2 years in stage 2 but does not prevent stage 3; and it does not halt progression to or reverse stage 2 to stage 1. Given its nonspecific anti-CD3 action, prolonged suppression of CD8 T cells relative to CD4 Tregs could potentially heighten malignancy risks over time.

## Secondary Prevention in Stage 1

The availability of generics and loss of patent protection render “nonapproved therapies” unlikely to gain formal approval. Nevertheless, off-label treatments, as supported by the American Academy of Pediatrics' policy statement (6), remain a viable option when guided by sound clinical judgment and supporting evidence. An example includes oral paricalcitol, a calcitriol analog, which has demonstrated promise in reducing T1D autoantibodies and mitigating disease progression across various stages, albeit with important acknowledged limitations (7).

## Primary Prevention Strategies

The feasibility of large-scale birth cohort studies, such as administering 2000 IU of vitamin D from birth, proven to reduce T1D risk by 78% within the first year (8), remains uncertain. Without pharmaceutical funding, conducting such randomized controlled trials poses significant challenges. Furthermore, withholding potentially preventive treatments, even when identified as potentially beneficial, raises ethical questions, particularly when education and psychological support alone may fail to adequately prevent complications such as diabetic ketoacidosis in real-world scenarios.

Rather than continuing perpetuate debates on the merits of vitamin D supplementation within established safety guidelines on daily doses that do not require medical supervision as in recommendation 2.6 from the Endocrine Society Clinical Practice Guideline, closely mirroring the upper limits defined by the Institute of Medicine, we could actively pursue primary prevention and expand research into potent calcitriol analogs as well as specific monoclonal antibody therapies and stem cell/B-cell immunotolerant regeneration therapies. Hippocrates, who emphasized the natural elements of health, likely understood the critical role of sunlight-dependent vitamin D synthesis—what he might be calling “Physis.” In the modern world, his ancient wisdom translates into the necessity of daily vitamin D supplementation to prevent illness.
